# An Analysis of the Mechanical Properties of the Ponseti Method in Clubfoot Treatment

**DOI:** 10.1155/2019/4308462

**Published:** 2019-03-25

**Authors:** Murtaza Kadhum, Mu-Huan Lee, Jan Czernuszka, Chris Lavy

**Affiliations:** ^1^Nuffield Department of Orthopaedics, Rheumatology and Musculoskeletal Science, Oxford University, UK; ^2^Department of Materials, Oxford University, UK

## Abstract

Congenital clubfoot is a complex pediatric foot deformity, occurring in approximately 1 in 1000 live births and resulting in significant disability, deformity, and pain if left untreated. The Ponseti method of manipulation is widely recognized as the gold standard treatment for congenital clubfoot; however, its mechanical aspects have not yet been fully explored. During the multiple manipulation-casting cycles, the tendons and ligaments on the medial and posterior aspect of the foot and ankle, which are identified as the rate-limiting tissues, usually undergo weekly sequential stretches, with a plaster of Paris cast applied after the stretch to maintain the length gained. This triggers extracellular matrix remodeling and tissue growth, but due to the viscoelastic properties of tendons and ligaments, the initial strain size, rate, and loading history will affect the relaxation behavior and mechanical strength of the tissue. To increase the efficiency of the Ponseti treatment, we discuss the theoretical possibilities of decreasing the size of the strain step and interval of casting and/or increasing the overall number of casts. This modification may provide more tensile stimuli, allow more time for remodeling, and preserve the mechanical integrity of the soft tissues.

## 1. Background

**Figure 1:**
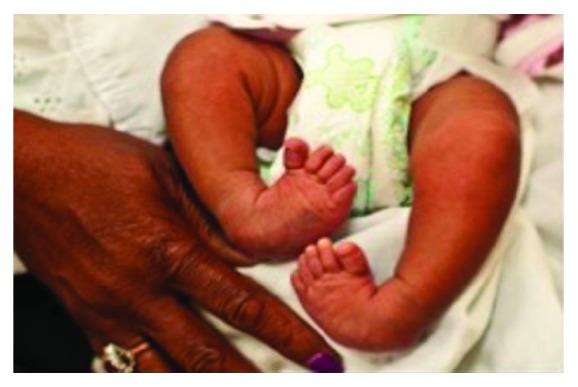
Bilateral clubfeet in a newborn infant. Image taken from CURE International with permission.

Congenital clubfoot or congenital talipes equinovarus (CTEV) is a complex pediatric foot deformity (Figure 1). It consists of four complex foot abnormalities with varying degrees of rigidity, namely, midfoot cavus, forefoot adductus, hindfoot varus, and hindfoot equinus [1, 2]. The incidence is widely reported as 1 in 1000 live births in the UK with males being affected about twice as often as females [1, 3]. In almost half of affected infants, both feet are involved. To date, the causes of clubfoot are poorly understood and regarded as idiopathic; however, genetic factors and associated conditions such as spinal bifida, cerebral palsy, and arthrogryposis have been reported [1, 3, 4].

If left untreated, clubfoot inevitably leads to significant long-term disability, deformity, and pain [2]. Although various surgical techniques are used to correct clubfoot, such as soft tissue releases or bony procedures in older children, currently, conservative management is the preferred option. Some surgical techniques have been shown to pose a greater risk of pain, stiffness, avascular necrosis, infection, overcorrection, poor long-term ankle range of movement, weakened mechanical strength, and arthritis than if treated conservatively [5–8]. Interestingly, some studies have also reported a correlation between the extent of release surgery and degree of functional impairment [6]. To date, surgical options are mainly employed to manage resistant cases and recurrence or if unable to achieve complete correction of the deformity.

Currently, the optimal treatment utilizes the Ponseti method, developed by Ignacio Ponseti in the 1940s [5, 9]. This technique consists of two distinct stages of manipulation and maintenance. The manipulation phase involves identifying the head of talus to use as a fulcrum, supinating the forefoot to eliminate the cavus deformity, and then abducting the forefoot. This manipulation is then followed up by application of a plaster cast, holding the foot in the corrected position and providing sufficient time for soft tissue remodeling. This manipulation-casting sequence is repeated on a weekly basis for an average of six weeks, until a 50-degree abduction of the foot around the tibia is achieved. An Achilles tenotomy may then be required to eliminate any residual equinus and is followed up by three weeks in a cast to aid healing in the lengthened position [1, 5, 9–11]. The maintenance phase then involves holding the foot in an abduction brace for 23 hours per day for 3 months, helping to reduce recurrence rates [10, 11]. Zionts et al. [12] reported that due to the increased use of the Ponseti method, the estimated percentage of clubfoot treated with surgical release has dropped from 72% in 1996 to 12%.

## 2. Main Text

### 2.1. Clubfoot Abnormalities

Due to the deformities, the dimension, structure, and mechanical properties of most soft tissues in a clubfoot are different to those of a normal foot. The presence of shortened, thickened, and fibrotic tissues at the medial and posterior aspect of the clubfoot has been reported in several studies [13, 14]. This includes thickening and shortening of the posterior tibial tendon, Achilles tendon, tibionavicular ligament (deltoid ligament), and plantar calcaneonavicular ligament. In addition, a fibrous matrix was also seen in the posterior fibulotalar and deltoid ligaments.

To our knowledge, no work on measuring the mechanical properties of the tendons and ligaments in a clubfoot by direct mechanical testing has been conducted. Masala et al. [15] investigated the difference in mechanical properties of the Achilles tendon between a clubfoot and a normal foot by real-time sonoelastography (RTSE). The results show lower mean elasticity values from the Achilles tendons of the clubfeet compared to normal feet (unilateral clubfoot patients), demonstrating that the Achilles tendon is stiffer in a clubfoot. Hattori et al. [16] compared the moduli of soft tissue on the medial, lateral, and posterior aspects of a clubfoot by a scanning acoustic microscope (SAM). They discovered higher Young's modulus for the calcaneofibular ligament compared to the deltoid ligament. This result implies that the lateral soft tissue contracture could also be responsible for some of the clubfoot deformities. However, the tissue samples used in this study were fixed in 4% paraformaldehyde before measurement, thus leading to excess crosslinking in the ligaments that would result in higher stiffness values. To note, in both of these studies [15, 16], the measurement of soft tissue elasticity was performed on patients undergoing or already treated with the Ponseti method. More information about the mechanical properties of the soft tissues in untreated clubfeet is needed for comparison.

### 2.2. Rate-Limiting Tissue: Tendon and Ligament

During the manipulation process of the Ponseti method, the soft tissues responding (or resisting) to stretching include the following: (1) skin, (2) capsule, and (3) tendons and ligaments. To investigate the main tissue that is restricting the foot from reaching the improved position during stretching, the stress values generated from the skin (*σ*_skin_), capsule (*σ*_cap_), and tendons and ligaments (*σ*_TL_) due to the mechanical stretch are required. To date, the stress values and elastic moduli (*E*_skin_, *E*_cap_, and *E*_TL_) in these soft tissues in response to stretching via the Ponseti method have not been studied.

**Figure 2:**
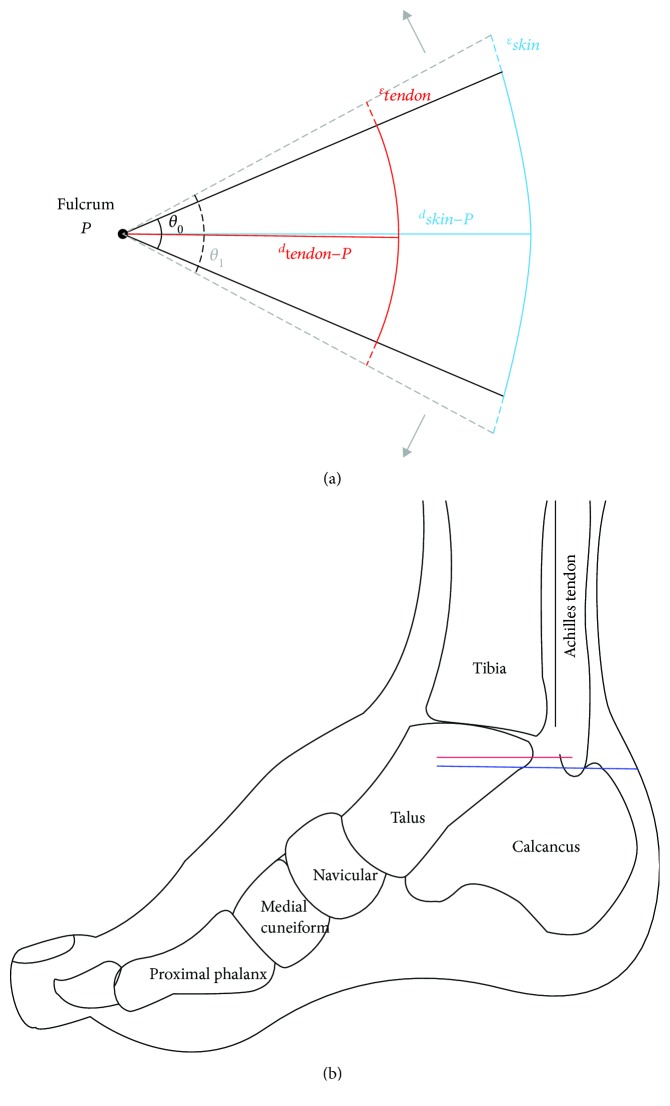
(a) Illustrative model of the deformations of the two different tissues (tendon and skin) due to a stretch with respect to a common fulcrum to correct equinus. The size of the force exerted to deform individual tissue is inversely proportional to the distance between the tissue and the fulcrum. (b) Diagram of the medial side of a foot with the red line indicating the talus-Achilles length and blue line indicating the talus-skin length.

First, consider a simple model simulating hindfoot dorsiflexion to correct the equinus deformity (Figure 2) in which a tendon tissue (red curve) and a skin tissue (blue curve) are located at distances of *d*_tendon−*P*_ and *d*_skin−*P*_, respectively, from the fulcrum (*P*). To introduce deformations of the tendon (*ε*_TL_) and skin (*ε*_skin_) by Ponseti manipulation, an angular change from *θ*_0_ to *θ*_1_ with respect to *P* is generated. Examining only the differences in the distance to the manipulation fulcrum between the two tissues, the forces needed on the tissues will be different to create the same angular change (or torque value). Based on the principle of leverage, a larger force is exerted on the tendon compared to the skin as the tendon is located closer to the fulcrum.

Second, the resistance of the tissues to stretch (stiffness) should also be considered. The general constitutive stress-strain relation can be described with the following:

(1) $$\begin{array}{c} \sigma =E\cdot \varepsilon . \end{array}$$

By calculating the product of Young's modulus of the tissue and the strain value produced by the stretch, the stress value required for manipulation can be acquired. A single stretch from a manipulation and casting will generate strain values (*ε*_skin_, *ε*_cap_, and *ε*_TL_) in each tissue. Typically, *ε*_skin_ and *ε*_TL_ will have approximately the same value, while *ε*_cap_ will be much smaller than *ε*_skin_ and *ε*_TL_ in any given stretch; hence, here we consider only tendons and ligaments and skin in our comparison. Young's moduli of human tendons and ligaments and human skin at the ankle from existing studies are listed in Table 1.

As Young's moduli of the tendons (lowest: 50 MPa) are larger than those of the skin (highest: 2 MPa) with almost similar tissue-fulcrum distances, larger stresses (or resistance) are generated from the tendons during the Ponseti treatment. Tendons and ligaments, therefore, are the rate-limiting soft tissues in the treatment of clubfoot.

## 3. Tendon and Ligament Mechanical Properties

### 3.1. Stress-Strain Curve

**Figure 3:**
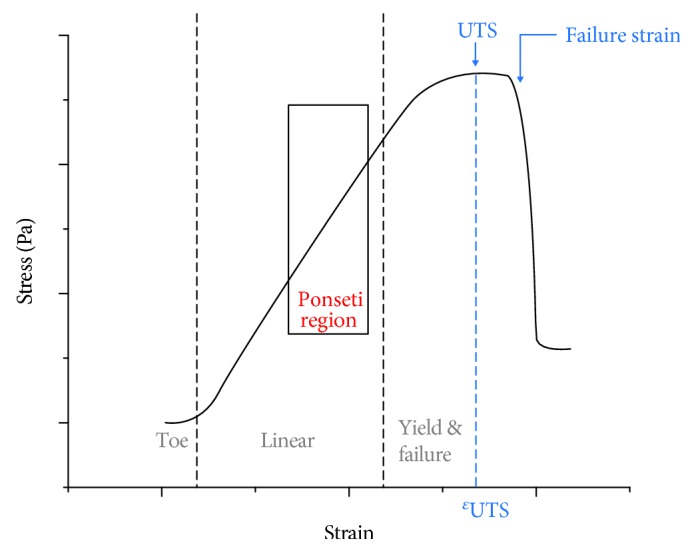
A typical stress-strain curve of a tendon or a ligament. Ultimate tensile strength (UTS) is the maximum stress that a material can withstand while being tensile loaded. Arbitrary values.

A typical stress-strain curve for a tensile test on a tendon or a ligament is demonstrated in Figure 3. The graph shows three distinct regions [28, 29]. Initially, the collagen crimps are stretched out, and an increasing number of collagen fibers and fibrils become aligned to the loading axis. This region is known as the “toe” region, and it normally extends to approximately 2% elongation [28]. The toe region lies within the elastic limit, and thus, the tissue will return to its original length when unloaded. Further straining brings the tissue into the “linear” region which exhibits constant Young's modulus. The stress-strain response mainly comes from elongating the aligned fibers and fibrils. Further straining induces plastic deformation by interfibrillar and interfiber sliding, and consequently, the tissue does not return to the original length and structure after unloading. In the last region (yield and failure region), in which macroscopic defects occur, yielding begins as the slope of the curve decreases, with inevitable tissue failure occurring with further load [28, 29].

**Figure 4:**
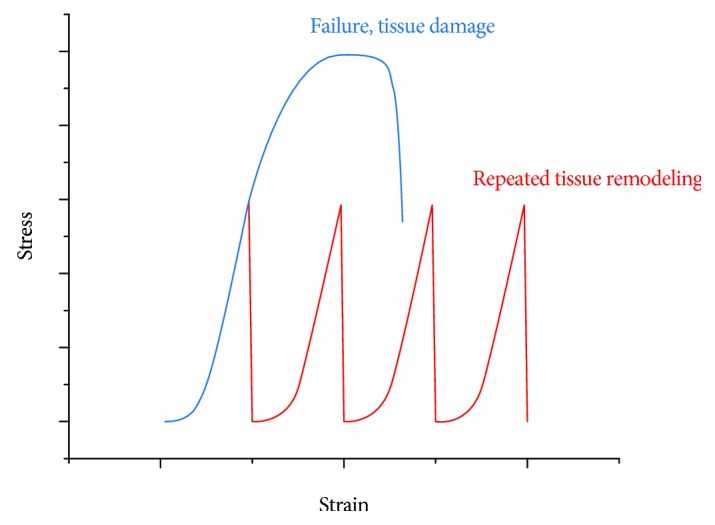
Comparison between a single tensile stretch (blue curve) and a Ponseti manipulation-casting sequence (red curve; sequential stress relaxation). Arbitrary values.

In the Ponseti treatment, the stretch caused by manipulation as aimed at producing sufficient plastic deformation of the tendons and ligaments to encourage tissue remodeling and lengthening. This deformation will normally lie within the middle part of the linear region (red bracket in Figure 3), as excess deformation would be painful and risk entering the yield and failure region, and insufficient deformation would prove ineffective. Notably, the total deformation needed to correct a clubfoot is greater than the failure strain in a single stretch, as displayed in Figure 4, proving that multiple stretches and castings are required in the Ponseti method. This factor is also clinically important, avoiding excess pain to the patient that may be generated in larger single stretches.

### 3.2. Ponseti's Loading: Stress Relaxation

A tendon or a ligament displays a time-dependent mechanical behavior known as viscoelasticity, which means it possesses both elastic and viscous properties [30]. Due to the viscoelastic behavior, tendons and ligaments display three characteristics: hysteresis, creep, and stress relaxation.

Creep describes the continuous increase in strain or deformation under constant loading force. The shape of the deformation-time curve during a creep test is dependent on the loading history (loading force, loading rate, and force increments) [31, 32]. Wren et al. reported that the time to failure decreases with increasing applied stress and increasing initial strain [33].

**Figure 5:**
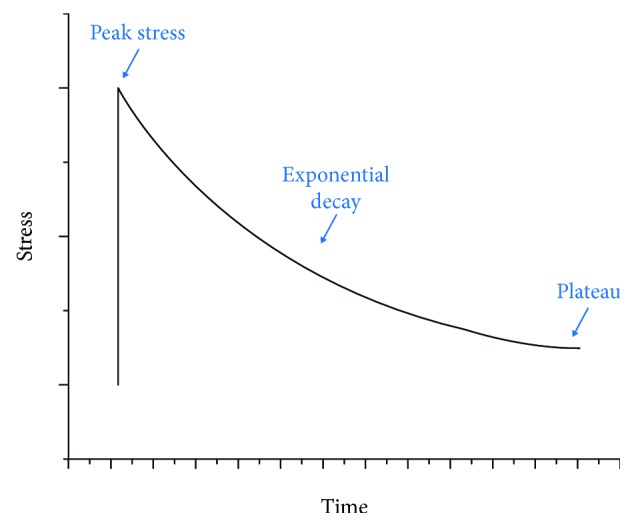
A typical stress-time curve of a single stress relaxation. Arbitrary values.

A stress relaxation, demonstrated in Figure 5, describes the continuous decrease in stress over time under constant strain. The relaxation rate of the stress is believed to be faster with a higher initial peak stress [34–36]. Under different strain rates applied to reach the initial strain, the tendon or ligament will display different relaxation profiles. With a higher strain rate, the corresponding peak stress will be higher, resulting a faster relaxation [31, 37].

It is worth highlighting that stress relaxation is an important event in the Ponseti method. As the clubfoot is held at an improved position by casting after manipulation, a constant strain or deformation is applied, and consequently, stress relaxation occurs in the strained soft tissues. The exponential relaxation is controlled by two events occurring in the tissues: (1) the microstructure rearrangement and (2) tissue growth. When stress relaxation begins, in response to the constant strain, the collagen fibers and fibrils start to reorganize themselves through interfiber and interfibrillar sliding controlled by the matrix molecules (proteoglycans). At the same time, recognizing these microstructural deformations, tissue remodeling causes subtle tissue growth, which in turn also contributes to the stress relaxation. The existing work of *in vitro* stress relaxation on tendons and ligaments only assesses the effect of microstructure rearrangement, without assessing the effect of tissue growth. It is thus reasonable to predict that the *in vivo* stress relaxation during a Ponseti stretch would be faster than the *in vitro* tests.

Multiple manipulation-casting cycles are required in the Ponseti method for effective clubfoot treatment, representing sequential stress relaxations on a stress-strain curve (Figure 4). To further study or improve the Ponseti method, measurements should be made to quantify the amount of the strain generated, the strain rate, and the corresponding peak stress in each casting. Rossetto et al. [38] discovered that both (1) a higher initial strain and (2) a longer relaxation duration would result in lower tensile strength (tested right after relaxation) in bovine calcaneal tendons. The relaxation rate was also faster in the higher initial strain group. Therefore, by introducing a smaller strain (smaller correction) or a shorter cast period for each casting phase, the stretched tendons and ligaments should be able to maintain stronger mechanical strength. Since the correction strain in each cast is smaller, the required number of casts has to increase in this modification. In ex vivo studies [36, 39–41], the time to plateau (relaxation time) for tendons is within an hour (2 minutes–1 hour). However, these experiments excluded the soft tissue remodeling (adaptation) which will occur *in vivo* during the treatment. In sum, the determination of the optimum cast-maintaining time needs consideration of the initial stain size, strain rate, relaxation time, and sufficient tissue remodeling.

### 3.3. Strain Rate Sensitivity

**Figure 6:**
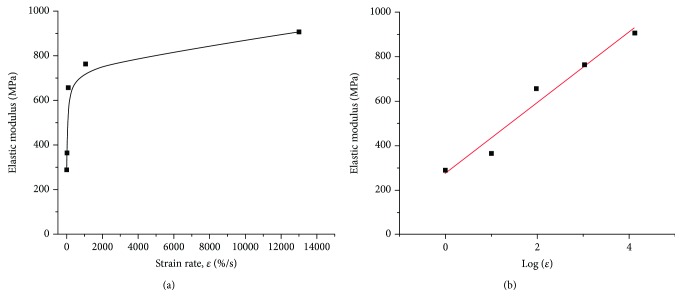
(a) Elastic modulus plotted against strain rate. (b) Elastic modulus plotted against the logarithm of strain rate [48].

The mechanical response of the tissue is dependent on the strain rate. In general, a faster loading results in a higher elastic modulus [42–45], as typically exhibited by polymers. Pioletti et al. [46] demonstrated that the bovine ACL displayed higher elastic moduli when stretched under higher strain rates (0.1, 1, 5, 10, 20, 30, and 40% s^−1^); in addition, the corresponding stress at the same strain value showed a positive correlation with strain rate as well. Woo et al. [47] and Danto and Woo [42] studied this effect under wider ranges of strain rates on medial collateral ligaments (0.011-222% s^−1^) and rabbit patellar tendons (0.016-135% s^−1^), respectively, and both showed the same positive correlation between elastic modulus and strain rate. According to the results from Bonner et al. [48], by plotting elastic modulus against strain rate, we can see a logarithmic relation between them (Figure 6). Hence, when a strain below a critical value of strain rate is applied, the mechanical properties of tendons and ligaments show stronger strain rate sensitivity. This critical rate could vary depending on the specific tendon or ligament tested, patient demographics, and comorbidities.

**Figure 7:**
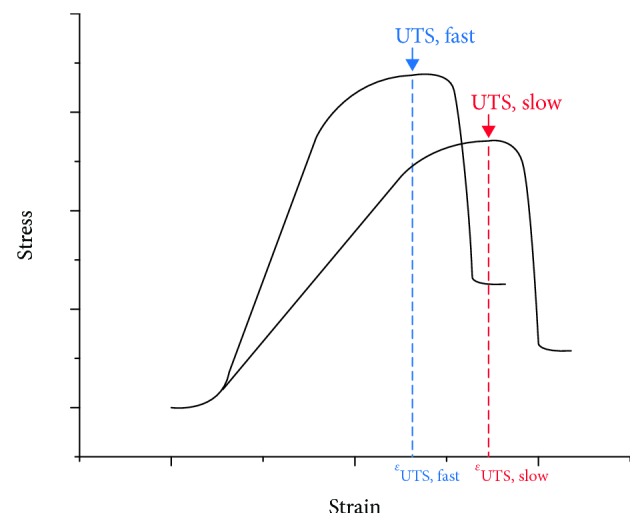
Stress-strain curves of a tendon or ligament stretched under a slow strain rate (blue) and a fast strain rate (red). Arbitrary values.

The increase in ultimate tensile strength (UTS)/failure stress and decrease in strain at UTS (*ɛ*_UTS_)/failure strain with increased strain rate were also reported (Table 2) [48–50]. These differences in mechanical response according to different strain rates can be seen in Figure 7.

An in situ X-ray study on rat tail tendons conducted by Bailey et al. [51] showed that the deformation of tendon is always larger than that of the individual fibrils, which suggests additional deformation in the ground substance matrix. In addition, the ratio of fibril deformation to tendon deformation increased as the strain rate increased in their experiment, i.e., the matrix becomes stiffer under higher strain rates. This phenomenon is believed to be caused by the decreased time for effective fluid flow, energy dissipation, and structural matrix rearrangement of the interfibrillar ground substance at higher strain rates [52], resulting in a smaller deformation of ground substance and a higher modulus tendon. Hence, further tension under high loading rates would cause the disruption of matrix-fibril bonding and poor transmission of stress between fibrils, which then lead to uncontrolled interfibrillar sliding and macroscopic failure [48, 51].

These studies on strain rate sensitivity of tendons and ligaments may help us understand and predict the stress-strain response in the biological system. For example, when a clubfoot is manipulated or stretched under a certain force, the expected strain or deformation of a tendon or a ligament can be designed by altering the strain rate. Furthermore, high strain rates, which are known to cause injuries (>50% s^−1^) and low *ɛ*_UTS_ (Table 2) [48, 49, 53, 54], should be avoided.

### 3.4. Mechanical Adaptation

Unlike synthetic materials, biological materials including tendons and ligaments are capable of adapting to chemical and physical stimuli. The cells in a tendon or a ligament, which express a fibroblastic phenotype, are responsible for responding to stimuli by producing growth factors [55], synthesizing collagen and ground substance of the ECM [56], and remodeling old ECM by releasing matrix metalloproteinases (MMPs) [57].

Mechanical loading or straining is a key stimulus which generates a series of signals responsible for maintaining, repairing, and modifying the ECM in tendons and ligaments. Fibroblast proliferation, morphology, alignment, and gene expression have been reported to be influenced by mechanical stimuli [58–60]. In terms of ECM synthesis and remodeling, studies have shown that cyclic tension on fibroblasts can increase collagen type I and collagen type III mRNA expression [60, 61]. These changes cause the number, diameter, and concentration of collagen fibrils to increase, thus increasing the stiffness of the tissue [62, 63]. By contrast, compression loading is believed to induce the synthesis of proteoglycans and type II collagen and, in some cases, a decrease in collagen type I [64–66]. This change in composition results in the formation of fibrocartilaginous matrix [64]. The adaptation of a tendon or a ligament is dependent on the type of loading, strain magnitude, strain rate, and number of cycles. It is worth mentioning that most existing studies on the effect of mechanical stretching on tissue adaptation applied short-term cyclic straining rather than static straining to the tissue [58–61, 67]. On the other hand, under a consistent strain rate or sequential stress relaxations for a longer period of time, the tissue response and adaptation will be different.

**Figure 8:**
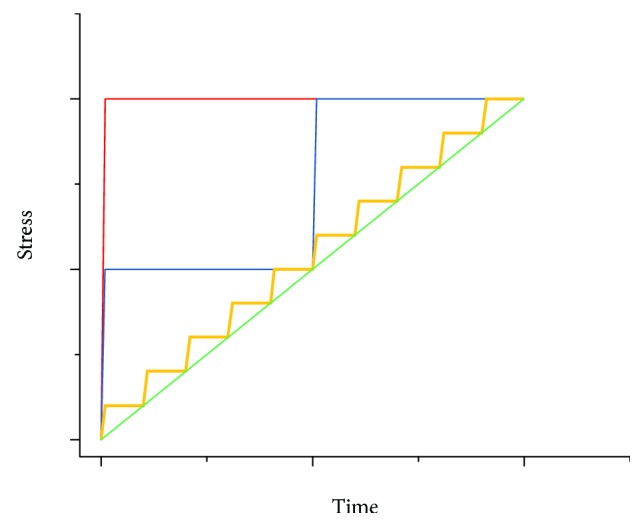
Incremental strain steps over time illustrating the sequential stretches in the Ponseti method performed in 1 step (red), 2 steps (blue), 10 steps (yellow), and infinite steps (green). Arbitrary values.

The Ponseti method utilizes a static stretch to a certain strain value during manipulation, followed by stress relaxation during casting. This stretch-casting sequence is repeated several times (sequential stress relaxations), thus generating a stepwise strain profile (Figure 8). Recent studies [68, 69] have shown that clubfeet with more severe deformities can be treated by increasing the number of castings. Nevertheless, the effect of the number of casts on the efficiency and correction rate of Ponseti treatment has not yet been investigated. In 2008, Screen [70] compared a single stress relaxation with sequential stress relaxations to the same amount of strain on rat tail tendons. Higher levels of interfiber relaxation, interfibrillar relaxation, and stress relaxation were observed in the single-step strained tendon. Lacking previous strain steps to reorganize the collagen structure, these single-step strained tendons are believed to have a higher risk of additional damage or loss of mechanical integrity.

**Figure 9:**
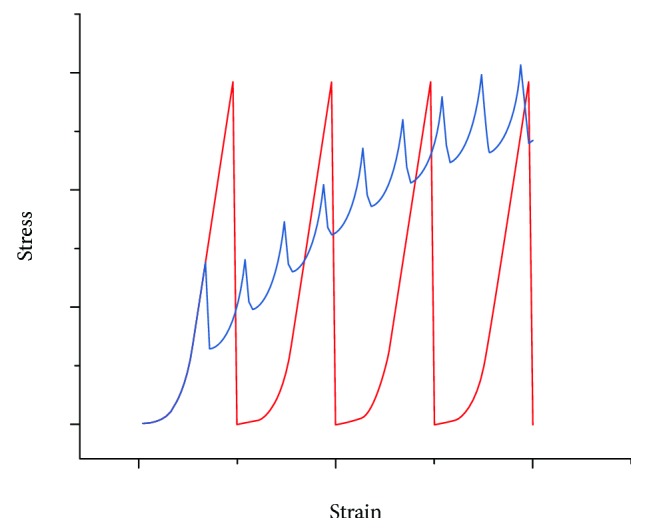
Illustrative stress-strain profiles of normal Ponseti sequential stretches (red), and a modified sequence (blue) with smaller strain steps, shorter casting duration, and a higher number of casts. Arbitrary values.

By decreasing the size of the strain step and increasing the numbers of steps (castings), the overall strain-time profile shown in Figure 8 appears to display decreasing slopes, i.e., a lower strain rate profile. This low strain-rate profile may provide the total correction process a larger *ɛ*_UTS_ (Figure 7). Furthermore, when more straining steps are applied, the overall stress-time profile shows higher stresses throughout compared with a single straining step [70]. A longer period of higher stresses maintained can be seen as “additional” tensile stimuli without overstretching the tissue. These gradual strain steps may also help the cells to “catch up” and remodel the ECM before considerable defects or ruptures occur.  Figure 9 compares the stress-strain curves between a normal Ponseti method and a modified version with more castings. In Figure 8, as the number of steps increases, the profile becomes comparable to a constant strain rate stretching. Although this approach would require more attendances by patients and shorter intervals between manipulations, it might improve the efficiency of the treatment. Kalson et al. [71] experimented the effect of static stretching on adaptation by slow stretching (0.25%/day) a tendon-like construct seeded with embryonic tendon cells for 4 days. The result showed increased collagen fibril diameter, fibril length, fibril number, and mechanical stiffness. The increase in the collagen fibril length and number is a promising indication of tendon growth or lengthening which is expected and desired in the Ponseti treatment. There are therefore possible potential benefits to treating clubfoot with a larger number of smaller, more frequent corrections.

**Figure 10:**
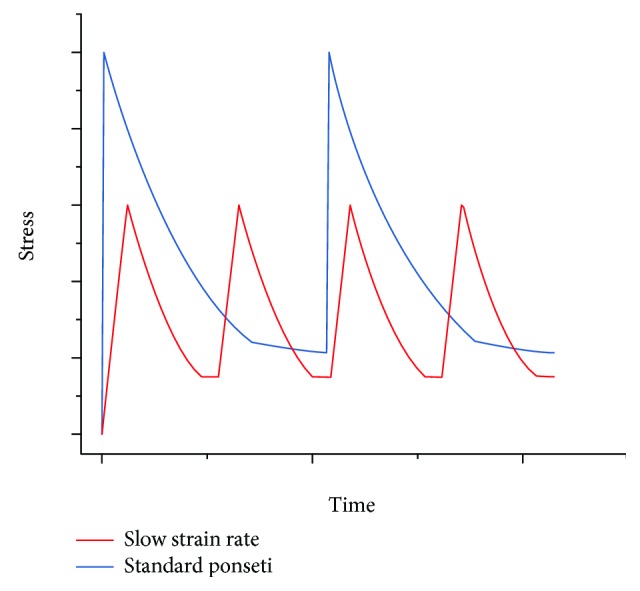
Stress-time profile comparing a standard Ponseti stress relaxation (blue) and a slow straining stress relaxation (red). Arbitrary values.

The efficiency of the treatment could also be improved by lowering the strain rate of the stretch before casting (first region before initial stress in Figure 5). As shown in Figure 10, under a lower strain rate during stretching, a smaller peak stress is generated in response to the same value of strain. Since the peak stress is lowered, the time required for stress relaxation (plateau) is also shortened, thus reducing the time interval between each cast. This modification allows the surgeon to produce the same degree of correction while reducing the casting period. However, some limitations do exist in this study. This paper is theoretical in nature and utilises arbitrary values derived from the literature. Some of these values were extrapolated from work investigating human adults, cadavers, or animal models. The recommendations derived from our model will require clinical correlation, which may be best achieved via a randomised controlled study.

## 4. Conclusions

The Ponseti method is widely recognized as the gold standard treatment for congenital clubfoot. During the multiple manipulation-casting series, the tendons and ligaments at the ankle, which are identified as the rate-limiting tissues, will undergo sequential stress relaxations. In each cast, the stretch brings the tendons and ligaments to the linear region of the stress-strain curve (Figure 3) generating sufficient plastic deformation and mechanical stimuli. Consequently, tissue growth or lengthening takes place by remodeling the extracellular matrix in response to the stretch. The main benefit of multiple castings (sequential stress relaxations) is the capability of reaching a high final strain (corrected foot) which is impossible to achieve in a single stretch (Figure 4). This additional tolerance of strain is attributed to the tissue adaptation process during the immobilization by casting.

Due to the viscoelastic properties of tendons and ligaments, the initial strain size, strain rate, and loading history will affect the relaxation behavior and mechanical strength of the tissue. Ideally, the manipulation should be performed as slowly as possible as under a lower strain rate shown in Figures 6 and 7, the tendon or ligament shows lower resistance due to lower elastic modulus and higher tolerance of strain (higher *ɛ*_UTS_). To increase the efficiency of the Ponseti treatment, we suggest consideration of decreasing the size of the strain step and interval of casting and/or increasing the overall number of casts. This modification may produce a lower overall strain rate profile (Figure 8), provide more tensile stimuli, allow more time for remodeling, and preserve the mechanical integrity of the soft tissues.

## Figures and Tables

**Table 1:** Elastic moduli of different human tendons, ligaments, and skin.

Soft tissue type	Source		Test method	Young's modulus (MPa)	Ref.
Human ankle tendons & ligaments	Plantaris tendon		*In vitro* tensile	1.24 × 10^3^	[17]
	Anterior tibialis tendon		Ultrasonography	0.45 − 1.2 × 10^3^	[18]
	Peroneus longus tendon		*In vitro* tensile	1 − 5 × 10^2^	[19]
	Peroneus brevis tendon		*In vitro* tensile	1 − 4 × 10^2^	[19]
	Calcaneal tendon		*In vitro* tensile	0.5 − 3.5 × 10^2^	[19]
	Calcaneofibular ligament		*In vitro* tensile	0.7 − 4.5 × 10^2^	[19]
	Achilles tendon		Ultrasonography	2 × 10^3^	[20]
	Deltoid ligaments		CT, MRI, and finite element modeling	0.45 − 2.7 × 10^3^	[21]
	Medial collateral ligaments		*In vitro* tensile	0.99 − 3.2 × 10^2^	[22]
	Lateral collateral ligaments		*In vitro* tensile	2.16 − 5.12 × 10^2^	[22]
Human skin	Neck		MRI and finite element modeling	~2	[23]
	Breast		Suction cup method	2 − 4.8 × 10^−1^	[24]
	Arm		*In vivo* tensile (extensometer)	1.3 − 6.57 × 10^−1^	[25]
	Arm		*In vivo* indentation	4.5 − 8 × 10^−3^	[26]
	Arm		*In vivo* indentation	~8.5 × 10^−3^	[27]

**Table 2:** Existing work demonstrating the strain rate sensitivity of tendons and ligaments.

Source	Strain rate (% s^−1^)	Elastic modulus (MPa)	UTS (MPa)	*ɛ*_UTS_ (%)	Ref.
Human Achilles tendon	10	401.1	73.3	25.2	[49, 50]
	100	544.7	81.3	21.0	
Source	Strain rate (% s^−1^)	Elastic modulus (MPa)	Failure stress (MPa)	Failure strain (%)	Ref.
Porcine lateral collateral ligament	1	288	39.9	17	[48]
	10	364	56.5	18	
	94	656	72.8	14	
	1060	763	75.9	11	
	12990	906	77.4	9	
